# Case report: Use of granulocyte-colony stimulating factor as an immunomodulatory therapy in a patient with neuromyelitis optica spectrum disorder and comorbid immunodeficiency

**DOI:** 10.3389/fneur.2023.1240356

**Published:** 2023-09-20

**Authors:** Gina S. Perez Giraldo, Edith L. Graham, Stephen VanHaerents, Roumen Balabanov

**Affiliations:** Neurology Department, Northwestern Medicine, Chicago, IL, United States

**Keywords:** neuromyelitis optica spectrum disorder, immunodeficiency, autoimmunity, granulocyte-colony stimulating factor, immunomodulation

## Abstract

**Background:**

Autoimmune diseases can coexist with immunodeficiency. We describe a treatment approach in which granulocyte-colony stimulating factor (G-CSF) is used to restore immune competence without worsening autoimmunity. G-CSF is a polyfunctional cytokine that influences survival, proliferation, and differentiation of hematopoietic stem cells, and has immunomodulatory effects on the innate and adaptive immune systems.

**Objective:**

To report a case of neuromyelitis optica spectrum disorder (NMOSD) with comorbid immunodeficiency and frequent infections.

**Methods:**

Case report and review of literature.

**Results:**

A 23 years-old man presented with a focal onset seizure with impaired awareness at age 12. At age 18, he developed headaches, recurrent multifocal seizures, and non-convulsive status epilepticus. Brain magnetic resonance imaging (MRI) showed extensive T2 hyperintense and gadolinium-enhancing periventricular and corpus callosum lesions. Serum aquaporin 4 antibody was positive 1:10,000 (normal value <1.5 titer), hence he was diagnosed with NMOSD. As a complication, patient developed mucormycotic pneumonia with cavitation, requiring thoracotomy precluding use of immunosuppressants. Gene testing demonstrated a mutation in MT-ND4 gene encoding for NADH dehydrogenase 4 in mitochondrial complex 1. Eventually, he began a treatment with filgrastim, a G-CSF analog, in addition to intravenous immunoglobulins and prednisone. Patient’s NMOSD has been in remission without relapses, or coexistent infections ever since.

**Conclusion:**

G-CSF is a polyfunctional cytokine with important immunomodulatory effects, which makes it an interesting therapeutic option when autoimmunity coexists with immunodeficiency and was used successfully in this case.

## Case presentation

**Figure 1:**
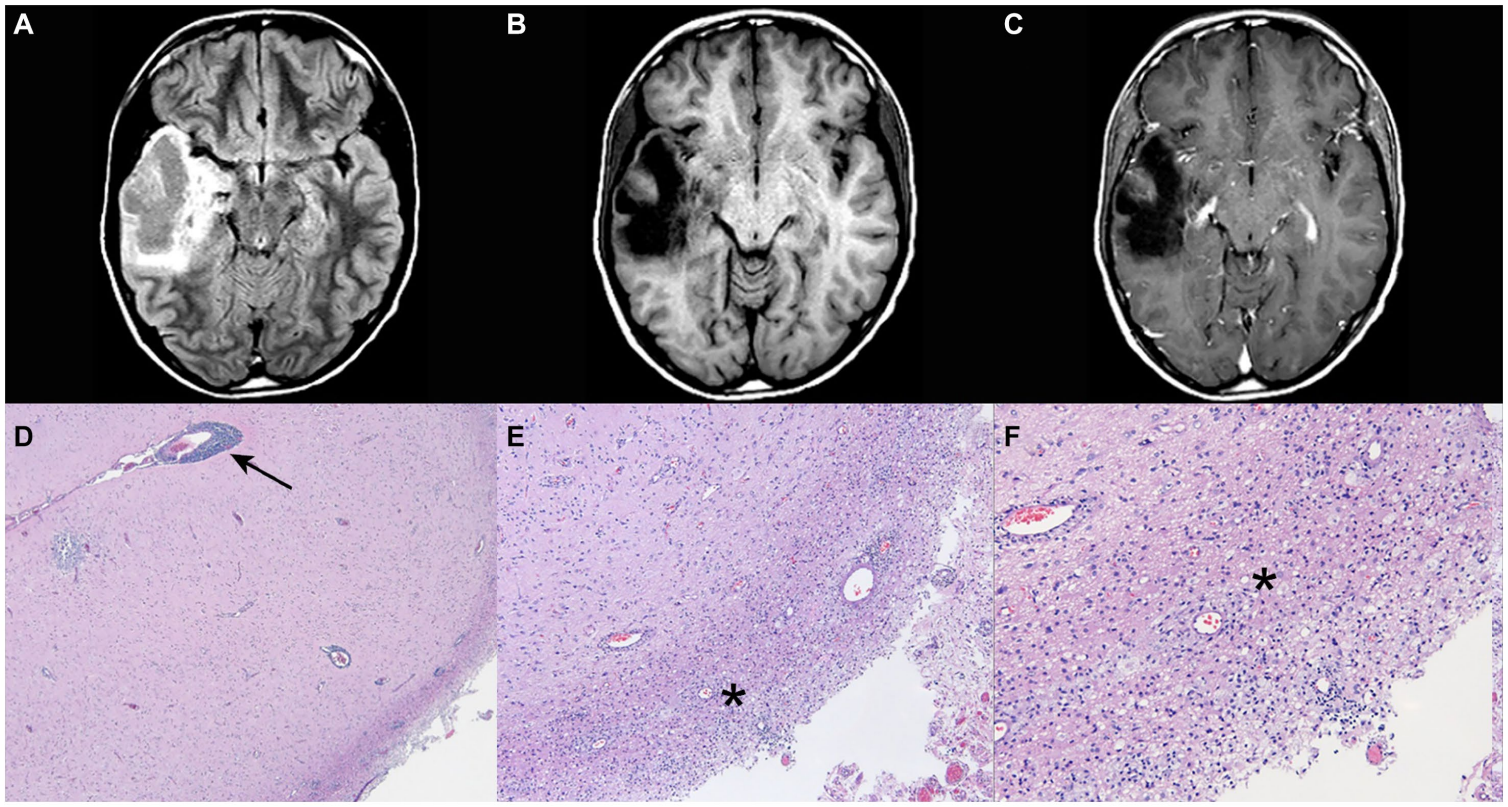
Post-operative magnetic resonance imaging (MRI) of brain with and without gadolinium and pathological findings at age 12. **(A)** FLAIR sequence. **(B)** T1 sequence. **(C)** T1 and gadolinium sequence (post lesion resection) with no evidence of contrast enhancement. **(D–F)** Perivascular lymphocytic infiltrates in the meninges and the cortex. There are geographic areas of dystrophic calcifications, cortical gliosis and degenerative changes in the white matter with cavitary features, and macrophage accumulation. Arrow points the area of perivascular infiltration and asterisks mark the parenchymal inflammation. Pathology images courtesy of Peter Pytel, MD, The University of Chicago.

A 23-year-old man presented initially with a focal onset seizure with impaired awareness at age 12. Magnetic resonance imaging (MRI) of brain obtained at the time of presentation demonstrated a right temporal mass lesion that was surgically resected soon after the first hospital admission (Figures 1A–C). Preliminary pathological report raised the question of low grade glioneuronal tumor based on morphologically abnormal neuronal cells, however, detailed subsequent examination revealed perivascular lymphocytic infiltrates in the meninges and cortex, with areas of dystrophic calcifications and gliosis, concluding that the lesion is due to an idiopathic inflammatory process (Figures 1D–F). After the surgery, patient remained seizure-free and off any anti-seizure drugs.

**Figure 2:**
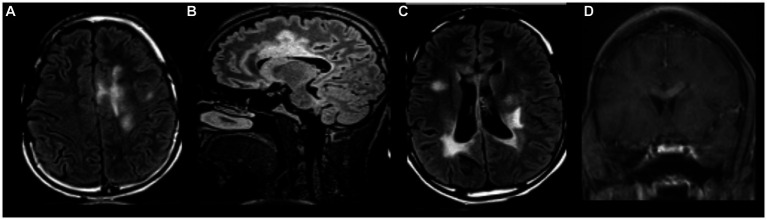
MRI brain with and without gadolinium at age 18. **(A–C)** Multifocal regions of white matter T2/FLAIR hyperintensity in both cerebral hemispheres and corpus callosum. **(D)** Gadolinium enhancement in corpus callosum.

**Figure 3:**
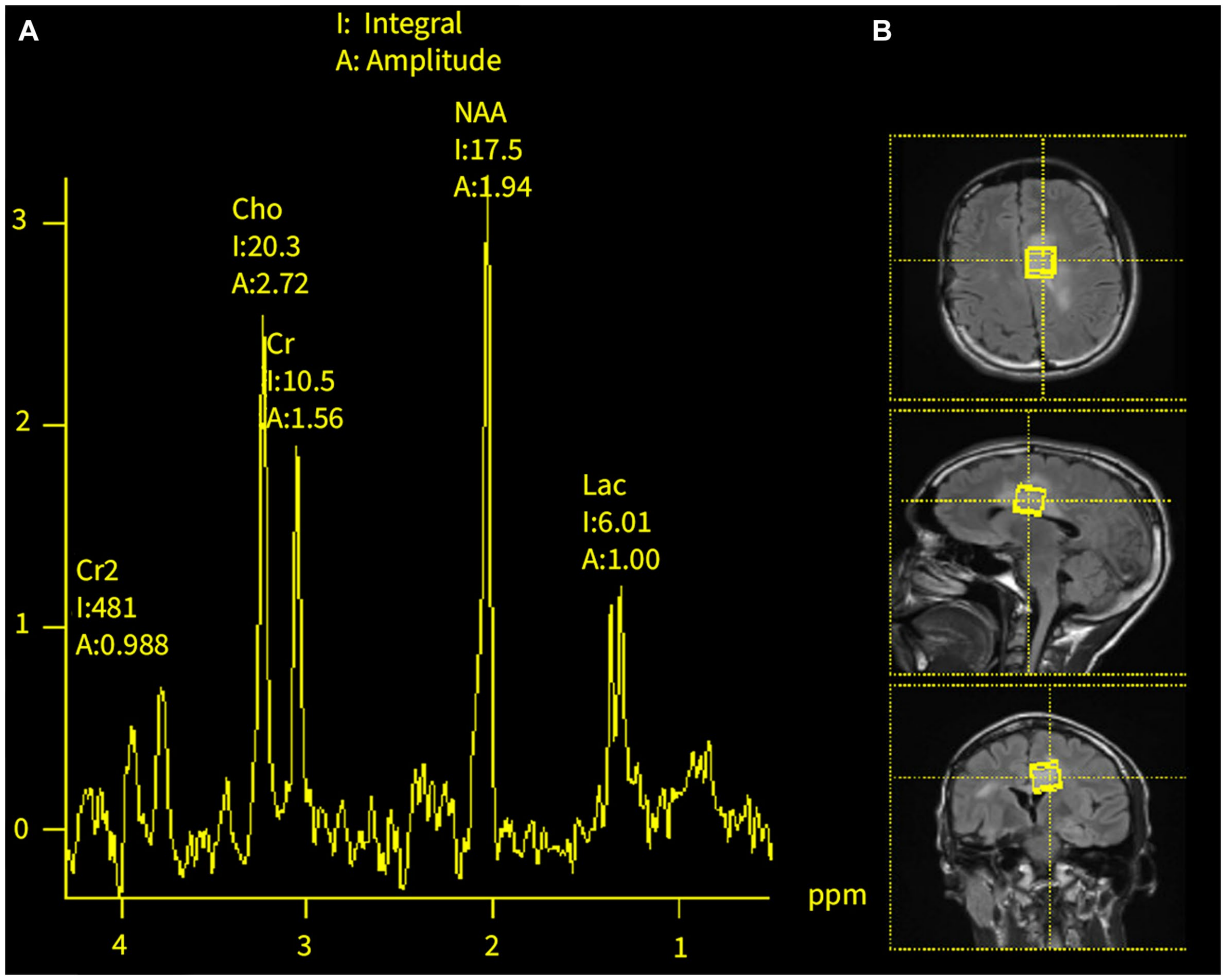
MR spectroscopy of brain lesions at age 18. **(A)** Double lactate peaks in the dominant T2/FLAIR hyperintense lesions of the corpus callosum and left cingulate gyrus. **(B)** Areas of MRS analysis.

At age 18, the patient developed headaches, recurrent multifocal seizures, and non-convulsive status epilepticus. Repeat brain MRI showed multiple gadolinium-enhancing lesions involving both hemispheres (Figure 2). Cerebrospinal fluid (CSF) analysis was unrevealing and non-indicative for a specific disease process. MR spectroscopy (MRS) analysis of one of the largest lesions detected a double lactate peak suggestive of a mitochondrial disease process (Figure 3). Combined Mito Genome Plus Panel (GeneDx) identified a mutation in MT-ND4 gene encoding for NADH dehydrogenase 4 in mitochondrial complex 1 (m.11985 A > G, heteroplasmy 37%). At age 19, patient was also diagnosed with neuromyelitis optica spectrum disorder (NMOSD) after the patient developed left eye optic neuritis and tested seropositive for anti-aquaporin 4 (AQP4) antibody (1:10,000 titer) (Supplementary Table S1).

Over the following 2 years, the NMOSD relapsed on several occasions, requiring rescue treatments, including methylprednisolone, plasmapheresis and rituximab. It was also noted that the patient developed slowly progressive hypogammaglobulinemia, leukopenia, neutropenia and lymphopenia. These hematological changes preceded the use of immunotherapy but further worsened following a single administration of 1 gram of rituximab. Importantly, these changes continued relentlessly, despite discontinuation of the aforementioned therapies reaching the point of severe immunodeficiency, and he suffered several common and opportunistic infections. On one occasion, the patient developed mucormycotic pneumonia with cavitation requiring pulmonary lobectomy, precluding further use of rituximab and other immunosuppressants. By age 22, patient had permanent neurological deficits, including complete visual loss in the left eye, right hemiparesis, and progression of lesion load on brain MRI, without clear immunotherapeutic options.

## Case resolution

Given the positive anti-aquaporin 4 antibody test, recurrent episodes of optic neuritis and tumefactive brain lesions, the patient was diagnosed with NMOSD. The MRS findings and genetic testing suggested a possible comorbid mitochondrial complex 1 disorder, which complicated the course of the disease, and likely predisposed the patient to leukopenia, neutropenia, lymphopenia and hypogammaglobulinemia. Patient was treated just once with plasmapheresis and rituximab, but the possibility of these therapies serving as a precipitating factor of the immunodeficient state can be also considered (1, 2). The life-threatening infections also precluded the use of the more recently approved medication by the Food and Drug Administration (FDA) for treatment of NMOSD.

Our neuroimmunology team faced a challenging clinical conundrum—coexistence of two progressive life-threatening disorders, one autoimmune in nature and the other associated with immunodeficiency, and with seemingly incompatible treatments. We designed a treatment strategy intending to restore protective immunity without triggering autoimmunity. The strategy included medications that have beneficial immunomodulatory effects and have been previously used in NMOSD (3–6). The medications included monthly administrations of intravenous immunoglobulin (IVIG) 0.4 g/kg (3, 4) (with the goal to normalize IgG levels in the blood, and as negative feedback for antibody production), oral prednisone 20 mg every other day (5) (as it has anti-inflammatory effects), as well as filgrastim (G-CSF) 300 mcg monthly (to stimulate myelopoiesis, lymphopoiesis and to maintain absolute neutrophil counts above 1,000 per microliter) (6). The patient also received carnitine, riboflavin, thiamine and arginine to ameliorate the negative metabolic effects of the comorbid mitochondrial disorder.

On this therapy, the patient, 25-year-old now, has been stable without NMOSD relapses or infections over the past 2 years. Therapy was well tolerated and not associated with significant side effects. The patient also became seizure-free with the resolution of the brain inflammation and the introduction of anti-seizure drugs.

## Discussion

As illustrated in our case, cytokines play an important role in neuro-immune interactions and are potential therapeutic targets in neurological diseases. NMOSD is an autoimmune inflammatory disorder of the central nervous system characterized by primary astrocytopathy, and bystander demyelination and neuronal injury. The disease was initially thought to be a rare variant of multiple sclerosis, but following the discovery of the antibody targeting the AQP4 channel, it became well accepted that NMOSD is a unique disease entity, with distinct pathophysiological features, a specific serum marker, clinical course and outcomes (7). AQP4 water channel is highly expressed by the astrocytic foot processes at the blood-brain barrier and by glial limitans of the subependymal regions of the brain (8). Upon binding to the AQP4, the autoantibody triggers a cascade-like inflammatory response characterized by the activation of the classical complement pathway and the recruitment of mononuclear and polymorphonuclear cells, all leading to astrocyte injury, downregulation of AQP4 expression, tissue swelling, cellular loss and structural damage (9). Interleukin 6 (IL-6) plays an important role in the disease pathogenesis as it promotes B cell proliferation and antibody production, facilitates the differentiation of Th17 cells and increases the permeability of the blood-brain barrier (9).

NMOSD is a rare sporadic disease of unknown etiology. Individuals with African and Asian descend are more susceptible to developing the disease, with an estimated prevalence of 10/100,000 and 3.5/100,000, respectively (10). Caucasians have an annual incidence of less than 1/1,000,000 and prevalence of 1/100,000. The disease is more common in women as compared to men, with a 9:1 ratio. Mean age of onset is around 40 years of age, however, children and the elderly can be affected as well (10). Clinical presentation of NMOSD includes unilateral or bilateral optic neuritis, affecting predominantly the posterior segment of optic nerve and chiasm, longitudinally extensive (more than 3 vertebral segments) transverse myelitis, and area postrema syndrome, associated with intractable hiccups, nausea and vomiting. As evident in our case, the cerebrum can be involved as well, sometimes with mass-like lesions, causing diagnostic challenges. Importantly, negative serum test for anti-AQP4 antibody does not exclude the diagnosis, even in less typical presentations. In fact, the current diagnostic criteria for NMOSD make it possible to reliably diagnose patients regardless the serostatus for the autoantibody (Supplementary Table S2) (11).

Currently, there are three FDA-approved monoclonal antibodies for the treatment of NMOSD: inebilizumab (anti-CD19), satralizumab (anti-IL-6) and eculizumab (anti-complement 5) (12–14). These medications are highly efficacious, with a reported reduction of attack risk of 77%, 74%, and 94%, respectively. However, these medications have significant immunosuppressive effects and carry strong time-dependent risks of infections, including meningococcal meningitis with eculizumab, neutropenia with satralizumab, hypogammaglobulinemia, hepatitis B and tuberculosis with inebilizumab; all precluding their use in our patient (12–14). Other medications such as rituximab (anti-CD20), mycophenolate mofetil, azathioprine, and methotrexate are sometimes used off label, as initially used in our case, but they are also immunosuppressive in nature.

Our patient with NMOSD posed a treatment challenge given the coexistent leukopenia, hypogammaglobulinemia, and recurrent life-threatening infections, possibly secondary to the mitochondrial disorder (15). The strategy of immunomodulation was effective in downregulating the autoimmune inflammation, without worsening the immunodeficiency, the treatment comprised of medications with both, broad immune supportive and anti-inflammatory properties, which in combination were able to sufficiently restore immune competence, avoid chronic and opportunistic infections, all without precipitating a NMOSD relapse. Filgrastim, which is a granulocyte colony-stimulating factor (G-CSF), played a pivotal role in our treatment strategy, not only due to its effects in leucocyte proliferation and differentiation, but also given its regulatory effect in the immune system, which is why G-CSF is considered a polyfunctional cytokine (Table 1) (16).

**Table 1:** G-CSF effects in the periphery and central nervous system.

Neutrophil proliferation and differentiation	Induces production of regulatory dendritic cells
Mobilization of progenitor stem cells from the bone marrow to the periphery	Reduces the production of inflammatory cytokines
Regulation of T cell activation	Reduces recruitment of T cells and IFN-γ secretion in CNS
T cell differentiation towards IL-4 and IL-10 producing phenotypes	Drives neuronal differentiation
Immune activation	Immunomodulation

Abbreviations: Il = interleukin, IFN-interferon.

G-CSF is a glycoprotein that can penetrate the blood-brain barrier and is encoded by the CSF3 gene. It is produced by hematopoietic and nonhematopoietic cells in all tissues within the human body via the activation of the JAK/STAT3 pathway (17, 18). G-CSF mobilizes progenitor stem cells from the bone marrow to the periphery and has immunomodulatory effects in the innate and adaptive immune system (17–19). Activated T lymphocytes and antigen presenting cells express functional G-CSF receptors, and exposure to G-CSF induces proliferation towards an IL-4 and IL-10 inducing T helper 2 rather than IL-2 and T helper 1 phenotype (17). Interestingly, CD4 T cells exposed to G-CSF acquire T regulatory cell properties, as they produce IL-10, IL-4, TGF-beta 1 and express FOXP3 (18). In addition, G-CSF inhibits T cell proliferation, maintains T cell hypo-responsiveness, and induces the production of regulatory dendritic cells in humans, which are antigen presenting cells important in the maintenance of self-tolerance (17–20). Finally, G-CSF can induce the expression of suppressor of cytokine signaling 3 (SOCS3), which is a regulator of T cell activation and differentiation, and reduces the production of inflammatory cytokines (18). Due to the immunomodulatory effect that G-CSF can have on the adaptive immune system, it is hypothesized that it could promote allograft tolerance after organ transplant (19).

In the central nervous system, neurons in all brain regions express G-CSF, including astrocytes (18). G-CSF has shown to reduce recruitment of T cells and IFN-γ secretion, limiting demyelination. It has also been implicated in neuroprotection via mobilization of autologous hematopoietic stem cells (HSC) to areas of cerebral damage after ischemic strokes, regulation of the differentiation of adult neuronal stem cells, activation of anti-apoptotic pathways and driving neuronal differentiation, ultimately promoting brain recovery (18). In the future, immunomodulation is likely to take the form of antigen-specific immunotherapy and induction of immune tolerance (21).

G-CSF also has immune-activating properties by promoting the production of lymphocytes, granulocytes and antigen presenting cells (APC), hence can aid in restoring the immune system after intense immunosuppression and has been used as a treatment strategy of progressive multifocal leukoencephalopathy (PML) in multiple sclerosis (MS) patients treated with natalizumab, by accelerating immune reconstitution inflammatory syndrome (IRIS) (6, 22).

Importantly, these reports indicate the existence of certain immunomodulatory properties of G-CSF that allow this cytokine to be used as an immune supportive agent without aggravating the autoimmune disease.

Finally, the association of NMOSD and mitochondrial disease is unknown and likely to be rare. Previously, MT-ND4 mutations have been linked to Leber’s hereditary optic neuropathy and multiple sclerosis, likely involved in the processes of neurodegeneration (23, 24). We are not aware of any reports implicating MT-ND4 gene mutation in NMOSD or immunodeficiency. Aquaporins 8 and 9 are expressed in the inner mitochondrial membrane and involved in the osmotic and volume regulation of the organelle (25). Interestingly, AQP4 deficiency disrupts cellular homeostasis and mitochondrial metabolism (26). Furthermore, anti-AQP4-mediated astrocytic damage leads to pronounced release of mitochondrial DNA in the central nervous system of NMOSD patients, which in turn, increases IL-1 production and probability of central nervous system inflammation (27). Hypothetically, downregulation of AQP4 expression in astrocytes might play a role in the integration and perpetuation of the mitochondrial and autoimmune disease processes.

In conclusion, our case highlights the need for further research on the role of mitochondrial dysfunction in NMOSD and the immune modulation as a treatment strategy in the management of complex cases in which immunodeficiency coexists with autoimmunity.

## Data availability statement

The original contributions presented in the study are included in the article/Supplementary material, further inquiries can be directed to the corresponding author.

## Ethics statement

Written informed consent was obtained from the individual(s) for the publication of any potentially identifiable images or data included in this article.

## Author contributions

All authors listed have made a substantial, direct, and intellectual contribution to the work and approved it for publication.

## Funding

GP’s fellowship was funded by the National Multiple Sclerosis Society.

## Conflict of interest

EG has received consulting and advisory board fees from Roche Genentech, Novartis, Atara Biotherapeutics, EMD Serono, Horizon Therapeutics, Tavistock Life Sciences, and American College of Physicians. She has received research support from F. Hoffman-La Roche Ltd. SV has received consulting fees from Atheneum Group, research support from the National Institute of Health and is involved in the ExTINGUISH trial. RB has received honoraria from Biogen, Sanofi, Alexion and Teva Pharmaceutical, and research support from the National Multiple Sclerosis Society, National Institute of Health, Nextcure, and Biogen.

The remaining authors declare that the research was conducted in the absence of any commercial or financial relationships that could be construed as a potential conflict of interest.

## Publisher’s note

All claims expressed in this article are solely those of the authors and do not necessarily represent those of their affiliated organizations, or those of the publisher, the editors and the reviewers. Any product that may be evaluated in this article, or claim that may be made by its manufacturer, is not guaranteed or endorsed by the publisher.
