# Supplementary material for: Case report: Use of granulocyte-colony stimulating factor as an immunomodulatory therapy in a patient with neuromyelitis optica spectrum disorder and comorbid immunodeficiency
Source: Front Neurol. 2023 Sep 20;14:1240356. doi: 10.3389/fneur.2023.1240356 (PMC10548877; doi:10.3389/fneur.2023.1240356)
Supplement: Supplementary file 1 [file Data_Sheet_1.PDF]

Supplemental Table 1. Pertinent diagnostic test results.

Diagnostic Testing	Results
MRI brain with and without gadolinium	T2/FLAIR hypertense lesions in subcortical white matter and corpus callosum associated with gadolinium enhancement
CSF analysis	WBC: 4 normal range: 0-5/µl<br>Glucose: 45 normal range: 40-70 mg/dL<br>Protein: 57 normal range: 15-45 mg/dL<br>IGG index: normal<br>OCB: negative<br>Lactic acid: normal<br>Negative tests for malignancy<br>Negative tests for JCV, VZV, HSV, EBV, CMV, T. pallidum, aerobic and Gram (+) bacteria and other causes of meningitis and encephalitis
EEG	Non-convulsive status epilepticus
MR spectroscopy	Double lactate peak suggestive of a mitochondrial disease process
Genetic testing	Mutation in MT-ND4 gene encoding for NADH dehydrogenase 4 in mitochondrial complex 1
Serum anti-AQP4 antibody	1:10,000 normal range: <1.5 titer
White blood cell count	WBC: 1.7 normal range: 3.6-10.8 x10^3µl<br>ANC: 0.8 normal range: 1.1-6.0 x10^3µl<br>ALC: 0.6 normal range: 0.7-3.4 x10^3µl
Immunoglobulin levels	IgA: 55 normal range: 66-433 mg/dL<br>IgG: 361 normal range: 635-1741 mg/dL<br>IgM: <12 normal range: 45-280 mg/dL
Serologies	Negative tests for HIV, T. pallidum<br>Normal tests for ESR and CR<br>SSA Ro: 8.0 normal range: <0.9 AI
Interleukin 6	8.63 normal range: <5pg/ml

Supplemental Table 2. Diagnostic Criteria for NMOSD

+ AQP4 IgG	- AQP4 IgG
1. At least 1 core clinical characteristic:<br>- Optic neuritis<br>- Acute myelitis<br>- Area postrema syndrome (intractable hiccups, nausea/vomiting)<br>- Acute brainstem syndrome	1. At least 2 core clinical characteristics, as a result of one or more clinical attacks, and:<br>- At least 1 core clinical characteristic must be optic neuritis, acute myelitis with LETM, or area postrema syndrome, AND<br>- Dissemination in space<br>- Fulfillment of additional MRI requirements

- Symptomatic narcolepsy or acute diencephalic clinical syndrome with MRI lesions<br>-Symptomatic cerebral syndrome with NMOSD typical brain lesions	
2. Positive test for AQP4 IgG	2. Negative AQP4 IgG test
3. Exclusion of alternative diagnosis	3. Exclusion of alternative diagnosis
